# Specialist involvement and referral patterns in ambulatory medical care for patients with dementia in Germany: results of a claims data based case-control study

**DOI:** 10.1186/1472-6963-11-148

**Published:** 2011-06-16

**Authors:** Hendrik van den Bussche, Birgitt Wiese, Daniela Koller, Marion Eisele, Hanna Kaduszkiewicz, Wolfgang Maier, Gerd Glaeske, Susanne Steinmann, Karl Wegscheider, Gerhard Schön

**Affiliations:** 1 Institute of Primary Medical Care, University Medical Center Hamburg-Eppendorf, Germany; 2 Institute of Biometrics, Hanover Medical University, Germany; 3 Division of Health Economics, Health Policy and Outcomes Research, Centre for Social Policy Research, University of Bremen, Parkallee 39, 28209 Bremen, Germany; 4 Department of Psychiatry, Psychotherapy and Medical Psychology, University of Bonn, Germany; 5 Institute of Medical Biometry and Epidemiology, University Medical Center Hamburg-Eppendorf, Germany

## Abstract

**Background:**

To analyze the referral processes from general practitioners to specialists and among specialists for dementia patients in the time periods before, during and after the diagnosis in Germany.

**Methods:**

In this case-control study claims data from 1,848 insurants with incident dementia aged 65 years and more and 7,392 matched controls were compared over a two-year period covering the pre-incidence, incidence and post-incidence time periods.

**Results:**

We found an increase in referrals of 30% in the incidence quarter, mainly from general practice to neuropsychiatry and from there to radiology. Referrals to clinical chemistry and other disciplines for dementia-specific reasons were negligible in amount. 34% of incident cases had at least one contact with a neuropsychiatrist during the year of incidence, and the majority of them visited this specialist repeatedly during that year. Only a minority (13.5%) of patients was referred to radiology for imaging. Referrals to other specialists declined whereas self-referrals did not increase.

**Conclusions:**

The referral rates to relevant specialists (neuropsychiatry, radiology and clinical chemistry) are far less frequent than proposed in German guidelines. More research is needed to explain the gape between guidelines and daily care and to find ways for a better implementation of guidelines in ambulatory care. Guidelines should not only deal with diagnostic procedures and therapeutic options but also consider questions of applicability in daily clinical practice and propose effective organizational models of care provision.

## Background

Referrals between primary care physicians (PCPs) and specialists are an important instrument in assuring the quality of ambulatory medical care, in Germany and elsewhere. In spite of the importance of referrals, the division of labour and the communication pathways between PCPs and specialists as well as between specialists of different disciplines have not been investigated thoroughly in health services research. Also, the quality of studies on medical referrals is generally low, as shown by Faulkner et al. [1] and by Akbari et al. in a recent Cochrane Review [2]. In Germany, only the Department of General Practice and Health Services Research at Heidelberg University has recently dealt with referral processes at the interface of primary and secondary ambulatory care, but in rather small-scale studies [3].

In order to understand the referral processes in dementia care, a basic understanding of the structure of ambulatory medical care services in Germany is necessary. The ambulatory medical services system in Germany is separate from the hospital sector. The ambulatory care sector consists of general practitioners and general internists acting as primary care physicians (PCPs) and an equal number of specialists solely operating outside the hospital setting, mainly in solo- or small group-practices. Among them, the disciplines of neurology and psychiatry (together neuropsychiatry - NP) are of special interest in the case of dementia. The average density of physicians in Germany was 1:1,433 population for PCPs and 1:14,361 for NPs in 2006, which corresponds to a factor 10 difference in supply [4]. All physicians involved in ambulatory care function as entrepreneurs in a market characterized by free access to all medical disciplines without compulsory gate-keeping. In daily life, however, the elderly heavily rely on their primary care physician (PCP), also for referrals. Since 2004, going to see the GP fist in a quarter is enforced by a co-payment of 10€/quarter when a specialist is contacted second in the quarter without a referral from the GP. Some 90% of the population is fully insured within a statutory health insurance scheme of the "Bismarckian" type [5].

Specific geriatric services hardly exist in Germany. In ambulatory care, only 0.3% of physicians hold a specific postgraduate training certificate for geriatrics. In the hospital sector this figure is 1.3%. Geriatric hospitals are also unequally distributed throughout the country [6]. The same applies to the some 100 outpatient memory clinics, usually affiliated to neurological, psychiatric or geriatric (departments in) larger hospitals, and only partially accredited and reimbursed by the statutory health insurance companies [7]. Thus, memory clinics play only a peripheral role in the treatment of dementia within the German statutory health insurance system. As a result, medical care for patients with dementia (including drug prescriptions, physiotherapy, occupational therapy, and medical services in nursing homes) is almost exclusively delivered within the regular ambulatory care sector described above. General hospitals play a role mainly for issues related to comorbidity (e.g. vascular problems) and/or dementia-related accidents (e.g. fractures), not for the treatment of the syndrome itself.

With regard to dementia, PCPs may have different reasons for referring to an NP in case of presumed dementia, e.g. for confirmation of diagnosis, differential diagnosis, assessment of neuropsychiatric comorbidity, and/or determination of the medication pattern. It is largely unclear, however, which criteria allow an optimal organization of the processes of referral, co-treatment and re-referral in case of dementia. This is partly due to varying opinions on the tasks of the PCP in relation to the specialist in dementia care [8,9]. Also, specifics of national health systems may influence the referral patterns, e.g. by determining who is authorized to prescribe specific drugs or by steering care pathways through gate-keeping and stepped-care concepts.

In a previous publication we showed a significant increase in the frequency of utilization of ambulatory medical services in Germany during the year prior to incidence, an increase nearly remaining unchanged in the year following incidence [10]. This increase in utilization applies to both the PCP and the specialist sectors. This increase in utilization concerned both the PCP and the specialist sector. Nearly all incident patients were seen by a PCP, whereas the percentage seen by an NP at least once during the year of diagnosis was 33%. But many questions remained. Which disciplines referred to the NP? To which extent did patients visit a specialist without a referral from a PCP? Did patients referred to an NP see this specialist only once (for diagnosis) or did they visit the specialist continuously over time? To which extent are other medical disciplines - e.g. radiology and clinical chemistry - involved in the diagnostic process as guidelines demand? Which medical disciplines refer to these diagnostic specialists? How do all of these disciplines and patients interact: Do these processes resemble a soccer game, in which the patient is "like a football passed back and forth between primary care physicians and specialist colleagues", as Feldman suggested in a recent editorial [11]?

To answer these questions a detailed analysis of the referral processes in ambulatory care was conducted using a large sample of patients with an ICD-coded diagnosis of dementia in conjunction with the German statutory health insurance system. The aim of this study was to analyze the driving forces and steering mechanisms in the referrals within the ambulatory medical care system for dementia patients in the time periods before, during and after the diagnostic process. Also, we investigated to which extent these referral processes are in accordance with guideline directives. The intention of the study was to detect possible deficiencies in the supply of services and/or the utilization of physician services for people suffering from dementia.

## Methods

The study is based on claims data for the years 2004 to 2006 from the Gmünder ErsatzKasse (GEK), one of the larger statutory health insurance company that operates nationwide and covers 1,7 million members. The observation period was one year before and one year after the first appearance of a relevant ICD-code (see below) in the records of a physician working within the statutory health insurance system in Germany. For historical reasons, the basic time period in the administration of the German healthcare system is not the year but the quarter. Claims are calculated on a quarterly basis, as are co-payment and referral regulations. As for research, this particular way of organizing data has the advantage of allowing detailed insight into long-term processes. The quarter in which the relevant ICD-code appears for the first time is called the incidence quarter. This administrative diagnosis is not necessarily identical with the first time a clinical diagnosis was made. The following criteria for inclusion were used:

• Age 65 and older.

• One ICD-10-code for dementia from the following list (F00.x, F01.x, F02.0, F02.3, F03, G30.x, G31.0, G31.1, G31.82, G31.9, and R54) in the records of a physician working in ambulatory medical care in at least 3 of 4 quarters within the one year observation period.

• An uninterrupted sequence of 4 quarters without an ICD-10-code for dementia before the first quarter with such a code ("incidence quarter").

• Uninterrupted insurance coverage in the GEK during the two observation years.

The control population was constructed by a 1:4 matching according to the nearest neighbour matching method of the MatchIt package for the "R" statistics software. Variables for matching were gender, year of birth, frequency of physician contact and number of visited physicians four quarters before the incidence quarter, whereby only the gender variable was required to be exactly matched. The two variables "frequency of physician contact" and "number of visited physicians" were chosen for matching because both groups should start from the same utilization figures in order to allow to observe differences afterwards. Further requirements for the control group were the absence of a dementia code and continuous health insurance within the 2 years of observation. To keep external influences as small as possible, the observation periods of the matched controls were chosen to be parallel to the ones of the dementia counterparts.

The main statistical analyses consist of multidimensional frequency tables with different percentages. Arithmetic means, standard deviations, and confidence intervals were conducted on all continuous outcomes. All analyses were performed using R statistics software.

Further details on the study design and the definition of the included specialist disciplines have been described in a previous paper [10]. The procedure of constructing the community types and the matching of abbreviated postal codes in order to analyze urban-rural differences has also been described elsewhere [12].

The study was approved by the Ethics Committee of the Medical Association of Hamburg.

With regard to the investigation of referrals, we first analyzed the referral processes during the incidence quarter since the vast majority of the referrals took place during this quarter, probably in order to secure the diagnosis. In a second step, we investigated the less frequent, additional referral processes in the quarters before and after the incidence quarter. We analyzed both referrals by different categories of physicians (rows 2 to 7 in table 1) as well as self-referrals. Self-referral takes place when a patient visits a specialist without a referral document from his PCP, a fully legal option in Germany (see rows 8 and 10 in table 1). Dementia-specific referral rates are obtained by comparing referral rates in the incident and control group in table 1. For reasons related to the database, as explained in the methods section of the previous publication [6], the referrals to specialized internists (cardiologists etc.) are excluded from the analysis. Therefore, figures on the total number of referrals are impacted by an estimated underreporting of 16% in the incident sample and 14% in the control sample. This may be a minor problem, as the above figures do not suggest a relevant increase of referrals to specialized internal medicine for dementia-specific reasons. The number of referrals in whatever discipline usually exceeds the number of referred persons because persons can be referred to more than one specialist physician by a referring physician within a given quarter. Also, a patient may visit one specialist on a referral basis and another one by self-referral. In both cases, two referrals are coded in the database for only one person in a particular quarter. A referral is valid for an entire quarter, and this implies that a patient can have more than one contact with the specialist in that particular quarter. Because of this database related difference between persons and processes, we distinguish between referred patients (=persons) and referrals. We present the data on the level of persons wherever possible. In some computations however (e.g. table 1) we had to use the case level for technical reasons.

**Table 1:** Size and direction of utilization of specialists* by type and source of referral in incident and control group in the incidence quarter

		Referred to neuropsychiatry (1)		Referred to radiology (2)		Referred to laboratory medicine (3)		Referred to other disciplines (4)		Referred to all disciplines together* (5)	
**Row no.**	**Type of referral**	**incident cases**	**controls**	**incident cases**	**controls**	**incident cases**	**controls**	**incident cases**	**controls**	**incident cases**	**controls**

1	Number of referrals by all disciplines (rows 2 - 7)	528	356	281	567	354	1145	896	3760	2059	5828

2	*Number (and %) of referrals by PCP*	*502 (95.1)*	*308 (86.5)*	*109 (38.8)*	*342 (60.3)*	*234 (66.1)*	*733 (64.0)*	*767 (85.6)*	*3253 (86.5)*	*1612 (78.3)*	*4636 (79.6)*

3	*Number (and %) of referrals by NP*	*8 (1.5)*	*5 (1.8)*	*136 (48.4)*	*35 (6.2)*	*29 (8.2)*	*8 (0.7)*	*13 (1.5)*	*15 (0.4)*	*186 (9.0)*	*63 (1.1)*

4	*Number (and %) of referrals by outpatient units*	*0*	*0*	*5 (1.8)*	*2 (0.4)*	*2 (0.6)*	*9 (0.8)*	*3 (0.3)*	*2 (0.1)*	*10 (0.5)*	*13 (0.2)*

5	*Number (and %) of referrals by radiology*	*0*	*0*	*1 (0.4)*	*5 (0.9)*	*5 (1.4)*	*6 (0.5)*	*2 (0.2)*	*6 (0.2)*	*8 (0.4)*	*17 (0.3)*

6	*Number (and %) of referrals by laboratory medicine*	*0*	*0*	*0*	*0*	*10 (2.8)*	*37 (3.2)*	*1 (0.1)*	*1 (0.0)*	*11 (0.5)*	*38 (0.6)*

7	*Number (and %) of referrals by other disciplines*	*18 (3.4)*	*43 (12.1)*	*30 (10.7)*	*183 (24.3)*	*74 (20.9)*	*352 (30.7)*	*110 (12.3)*	*483 (12.9)*	*233 (11.3)*	*1061 (18.2)*

8	Number of self-referrals	105	76	6	15	6	25	306	1068	423	1184

9	Total number of utilizations (rows 1 + 8)	633	432	287	582	360	1170	1202	4828	2482	7012

10	Self-referrals in % of all utilizations (8 in % of 9)	16.6	17.6	2.1	2.6	1.7	2.1	31.5	25.5	17.0	16.9

* without referrals to specialized internists; PCP = primary care physician, NP = neuropsychiatrist

Reading example: 528 referrals by a physician and 105 self-referrals to neuropsychiatry were found in the incidence quarter; 502 referrals of 528 were done by a primary care physician (95.1%).

The number of referrals is 3% higher than the number of referred persons (see methods chapter)

## Results

### Socio-demographic structure of the sample

The cohort consists of 1,848 persons (mean age 78.72 years with range 65 to 102 years, percentage of women 47.6) with an incident diagnosis of dementia and 7,392 controls (mean age 78.74 years with range 65 to 100 years, percentage of women 47.6). As described in a previous paper [10], patients with incident dementia had on average 12.4 contacts with physicians in the incidence quarter, some 50% more than controls (8.3 contacts; p < 0.001). These contacts were distributed over 3.1 different physicians in the incident sample versus 2.4 physicians in the control sample (p < 0.001). The amount of physician contacts grew steadily during the year prior to the incidence quarter and remained high during the year after the first coding of the diagnosis.

### Referrals in the incidence quarter

Table 1 shows the referrals from (rows) and to (columns) the most important medical disciplines as well as the proportions of physician-ordered referrals and self-utilization of specialists in the incidence quarter. Analyses apply to controls with at least one physician contact within this quarter. Therefore, the number of controls is reduced to 6772

The results in table 1 can be summarized as follows:

• Overall referral picture: The incidence quarter shows increased overall referral activity as the average number of physician-ordered referrals is 0.79 per person for controls and 1.11 for incident cases (+40.5%). During the incident quarter, 78% of all referrals in the incident sample and 80% in the control group were made by the PCP (column 5, row 2), which makes this discipline - as expected - by far the main referring discipline within ambulatory medical care. PCPs themselves are visited by nearly all (95%) incident cases and controls without a referral.

• Referrals to neuropsychiatry: 34.3% of incident cases and 6.4% of controls visited an NP during the incidence quarter (row 9; column 1; p < 0.001). Together, these figures means that 27.9% of the incident cases are seen by an NP for dementia-specific reasons during the incidence quarter. 95.1% of the physician-ordered referrals of incident cases to a NP were made by a PCP (row 2, column 1), and 16.5% of all referrals were self-referred. Compared to controls, the incidence of dementia did not increase the number of self-referrals (row 10, column 1).

• Referrals to radiology: 15.5% of incident cases and 8.6% of controls (p < 0.001) were referred to radiology, which corresponds to a referral percentage of incident cases for dementia-specific reasons of 6.9%. PCP exhibited nearly no increase in their referral rate to radiology (5.9% of incident cases vs. 5.1% for controls; row 2; column 2) whereas NPs referred a much larger proportion of their patients to radiology (25.8% of incident cases vs. 9.8% of controls; row 1, column 1 vs. row 3; column 2). As a result, the referral rate of NPs to radiology in the incidence quarter was 16% of all dementia-specific referrals to an NP.

• Referrals to clinical chemistry: A referral to clinical chemistry and other laboratory disciplines (row 1, column 3) takes place in only 2.3% of incident cases for dementia-specific reasons (19.2% for incident cases vs. 16.9% for controls; p < 0.001). PCP ordered 66.1% and NPs 8.2% of the referrals to clinical chemistry for incident cases (column 3, rows 2 and 3).

• Referrals to other medical disciplines: The referral rate to all other medical disciplines is slightly lower in the incident sample than for controls (48.5% vs. 55.5%; see column 4; p < .0001). On the other hand, the absolute number shows that more than half of the referrals in the incidence quarter (probably) go to specialists for other reasons than dementia.

• Referrals to outpatient memory clinics: As stated earlier, the data on the utilization of outpatient units (memory clinics) are affected by under-reporting as the visits to these institutions are not reimbursed by all statutory health insurance companies. The most important finding not affected by underreporting is that 75% of those statutory insured patients who attended memory clinics were self-referred patients. This suggests a highly selected patient clientele in German memory clinics.

### Patterns of utilization and referral before and after the incidence quarter

Diagnostic and/or treatment activities by specialists may of course also take place in the quarters before or after the incidence quarter. Therefore, we also analyzed the specific patterns of utilization and referral over the 2-year observation period (quarters 1 to 4 before incidence quarter and quarters 5 to 8 consisting of incidence quarter plus the 3 following quarters). Table 2 shows the representation of typical patterns in the incident group as compared to controls. These patterns were selected for exemplary reasons:

**Table 2:** Patterns of utilization of specialist disciplines in incident and control group over the two-year observation period*

		Neuropsychiatry pattern percentages		Radiology pattern percentages		Laboratory medicine pattern percentages	
**Pattern number**	**Pattern description**	**incident group**	**control group**	**incident group**	**control group**	**incident group**	**control group**

1	Contacts over 3 or 4 quarters in quarters 5 to 8	54.9	26.3	2.9	5.0	14.5	16.9

2	Contact in only 1 quarter in quarters 5 to 8	23.1	55.2	79.8	78.0	59.5	61.2

3	Two contacts or more in quarters 1 to 4	35.6	41.6	22.8	23.6	37.0	39.9

4	Two contacts or more in quarters 1 to 4 and in quarters 5 to 8	24.7	9.4	2.0	2.7	10.6	12.0

* Numbers refer to patients with at least one contact with a specific discipline

Quarters 1 - 4 = quarters before incidence/diagnosis quarter; quarter 5 = incidence/diagnosis quarter, quarters 6 - 8 = quarters after incidence/diagnosis quarter

• Pattern 1 stands for continuous specialist care in the year after diagnosis, but not in the year before diagnosis.

• Pattern 2 represents single specialist contact in the year after diagnosis.

• Pattern 3 stands for multiple contacts with a specialist in the year before diagnosis but none in the year after.

• Pattern 4 represents continuous specialist care in the year before and after diagnosis.

Table 3 describes the same issue of continuity in the form of the "losses" and the "gains" of patients by NPs in the quarters after the incidence quarter. The table shows how many patients referred to a specialist discipline visit this specialist in which quarter and how many stay with this specialist in the quarters thereafter.

**Table 3:** Quarter of first contact and course patterns of incident patients visiting an NP (n = 831) and/or a radiologist (n = 538) during the incidence year

	Row nr.	Quarter 5	Quarter 6	Quarter 7	Quarter 8
Course patterns for neuropsychiatry	1	612 (100%)	486 (79%)	405 (66%)	352 (58%)
	2		100 (100%)	61 (61%)	51 (51%)
	3			72 (100%)	37 (51%)
	4				47 (100%)

Course patterns forradiology	5	275 (100%)	39 (14%)	6 (2%)	3 (1%)
	6		126 (100%)	10 (8%)	3 (2%)
	7			71 (100%)	9 (13%)
	8				66 (100%)

Quarters 1 - 4 = quarters before incidence/diagnosis quarter; quarter 5 = incidence/diagnosis quarter, quarters 6 - 8 = quarters after incidence/diagnosis quarter

Reading example for NP: 831 incident patients visited an NP for the first time in single quarters of the incidence year [612 in IQ 5 (incidence quarter), 100 in IQ 6, 72 in IQ 7 and 47 in IQ 8]. These first-time-visit groups (all set at 100%) are followed during the next quarters: among the 612 patients of Q 5, 79% see an NP again during Q 6 (and 21% drop out), 66% also in Q 7, and 58% also in Q 8. Of the 100 first-time visitors in Q 6, 61% see an NP again during the next quarter (IQ 7) etc.

The results of tables 2 and 3 can be summarized as follows:

• Involvement of neuropsychiatry: Adding together the figures of table 1 and 3, we found that a total of 33.4% of the incident patients visited an NP for dementia-specific reasons during the year of incidence (incidence quarter + 3 quarters). 79.6% of the incident cases visiting an NP in the course of the incidence year did so in a relatively continuous manner over several quarters (see pattern numbers 1 and 4 in table 2), and 24.7% were seen by the NP in a continuous manner over the two-year period, whereas this is only the case for less than 10% of patients consulting for other reasons than dementia (pattern 4 only). Only a minority (23.1%) visited the NP only once in the quarters after diagnosis (pattern number 2). This pattern is clearly distinct from the utilization pattern of the controls visiting an NP for other reasons than dementia, where the majority has only one contact over 12 months.

The large majority of those who maintained a continuous contact with a NP were referred every quarter by their PCP. Self-referral did not increase for those cases. This continuous pattern of care is inversely confirmed by pattern number 2, which stands for a discontinuous specialist care pattern after diagnosis: Less than one fourth of the incident cases can be classified in this group, whereas the inverse is the case for controls (p < 0.001). For 28% of the patients, irregular patterns were found that prevented interpretation.

Table 3 shows that 79% of the patients who visited an NP during the incidence quarter visited the NP at least once during the subsequent quarter, and this relationship remains remarkably stable as only 13% resp. 8% of the patients refrained from visiting the NP during the next two quarters. The vast majority (74%) of patients who visited an NP during the diagnosis year did so for the first time already during the diagnosis quarter. Like table 2, table 3 shows that more than half of the patients stay with an NP over (at least) a period of one year.

• Involvement of radiology: Table 2 shows that the usual contact pattern with radiology is one contact only in one of the post-incidence quarters. Table 3 confirms this conclusion but shows also that half of the patients who visited a radiologist within the year of diagnosis were examined in the incidence quarter, the other half in the following quarters. Table 2 also shows that only a small number of incident cases were examined in a pre-incidence quarter. Putting together the figures of table 1 and 3, we conclude that 20.5% of the incident cases visited a radiologist for dementia-specific reasons during the year of incidence.

• Involvement of clinical chemistry: Table 2 shows that the patterns of utilizing laboratory medicine over the two years of observation do not differ between the incident and the control group. It can therefore be concluded that the incidence of dementia does not lead to an increasing demand for laboratory examinations. The majority of contacts is of the one-quarter type, for incident cases mainly during the diagnosis quarter or thereafter.

### Influence of referrals on diagnosis

The interplay between PCPs and NPs in the diagnosis process of dementia raises two main questions:

- Which profession made the first diagnosis and which diagnoses are made?

- What happened to a first diagnosis made by a GP in the incidence quarter when at least one visit to an NP (and not to another diagnosing physician) took place during the next 3 quarters (n = 120)?

In 60.4% of cases, the initial diagnosis was made by a PCP only and in 8.6% by a NP only. In 16.0% of cases, the diagnosis was made by both disciplines jointly (see table 4). Table 4 also shows, however, that the relative distribution of codes differs extremely from that found in epidemiological incidence research (see discussion section). This is not only the case for the diagnoses made by the GP but - to a lesser extent - also by the NP as almost half of the diagnoses made by the NP alone are classified as dementia unspecified.

**Table 4:** Sources and types of dementia diagnoses in ambulatory care in the incidence year (n = 1848)

	Source of dementia diagnosis					
**Type of diagnosis**	**All (%)**	**PCP only**	**NP only**	**PCP + NP**	Other discipline only*	Several other disciplines*

Alzheimer dementia	143 (7.7)	88 (7.9)	26 (16.5)	18 (6.1)	4 (7.1)	9 (4.2)
Vascular dementia	257 (13.9)	202 (18.1)	35 (22.2)	13 (4.4)	5 (8.9)	2 (0.9)
Specific dementia	15 (0.8)	8 (0.7)	3 (1.9)	1 (0.3)	3 (5.4)	0 (0.0)
Unspecified dementia	927 (50.2)	689 (61.7)	73 (46.2)	65 (22.0)	41 (73.2)	59 (27.3)
Mixed diagnoses	506 (27.4)	129 (11.6)	21 (13.3)	207 (69.9)	3 (5.4)	147 (68.1)
All	1848 (100)	1116 (100)	158 (100)	296 (100)	56 (100)	216 (100)

PCP = primary care physician, NP = neuropsychiatrist * except PCP and/or NP

Reading example: among all diagnoses 7.7% were classified as Alzheimer's disease and 13.9% as vascular. Among those who received their diagnosis from the PCP only, 7.9% were classified as Alzheimer's disease.

When the diagnosis was first made by the GP and the patient visited an NP once or more during the subsequent quarters, the original diagnosis made by the GP was confirmed by the NP in exactly 50% of the cases, while the most frequent alteration of diagnoses was a change from unspecified dementia to Alzheimer's disease (35.7% of the originally "unspecified" GP-diagnoses).

### Factors influencing referrals

We investigated the influence of age, gender and rural-urban differences on the utilization of neuropsychiatry and radiology. We performed logistic regression analyses for the following outcomes: contact with NP in the incidence year yes/no, contact with NP in at least 3 quarters of the incidence year vs. contact in 1 or 2 quarters, contact with radiology in the incidence year yes/no. For contacts with NP an influence of gender was not found (OR = 0.93; CI: 0.76 - 1.13, and OR = 0.96; CI: 0.72 - 1.29 respectively). As for age, every year older decreased the chance to visit an NP in the incidence year by 7% (OR = 0.93; CI: 0.92 - 0.94) and the chance to visit the NP continuously by 2% (OR = 0.98; CI: 0.96 - 1.00) when controlled for other factors. The chance to contact an NP when living in a rural community was 36% lower (OR = 0.64; CI: 0.52 - 0.79) and the chance for continuous visits 41% lower (OR = 0.59; CI: 0.42 - 0.82) than in an urban area. The chance to visit a radiologist in the incidence year was also gender independent (OR = 0.96; CI: 0.77 - 1.19), decreased also with age (OR = 0.92; CI: 0.90 - 0.93), but was independent of the regional setting (OR = 0.93; CI: 0.74 - 1.18).

## Discussion

Not surprisingly, this study confirms the crucial role of the PCP in the care of patients with dementia in Germany. As shown in a previous paper, patients with dementia have substantially more contact per quarter with their PCP than controls [10]. In this subsequent paper we show that the PCP is also the most important steering source in ambulatory medical care, as most referrals to specialists are made by PCPs.

Overall, the relative number of referrals increases substantially (some 30%) in the incident sample during the incidence quarter. The substantial increase of referrals for dementia-specific reasons to neuropsychiatry and - to a lesser extent - to radiology is somewhat counterbalanced by a slightly reduced rate of referrals by and to dementia-unspecific disciplines, suggesting that dementia is - as expected - the major medical concern during the incidence quarter for the PCP (and probably for the patient). This shift of referrals in the diagnosis period was also found in a cross-sectional study by Schubert et al. based on a regional dataset of another German health insurance company in 2002 [13]. The large proportion of referrals going to dementia-unspecific disciplines, even during the incidence quarter, point to the many comorbidities of these patients which need further specialist care according to German standards [14].

Most of the dementia-specific referrals go - as expected - to NPs. Under consideration of the utilization rate of the controls, the percentage of incident cases visiting an NP for reasons of dementia during the year of incidence was 33.4%. This number includes both the referred patients and those who consult an NP without referral. This percentage is slightly higher than the 30% found by Schubert et al. [13]. Whereas gender did not play a role, the patients referred to the NP (and to radiology) were relatively younger, a finding confirming the study of Lopponen et al. for Finland [15]. For France, the Trois-Cités-study also found that a non-referral to specialist care was higher for the oldest and the less educated patients. These authors also stressed that the barriers to referral between primary and secondary care may lay both in the patients and the GPs [16]. The referral rate may also depend on the readiness of the relatives to comply to a referral and of the specialists to accept such patients [17].

In fact, with regard to specialist dementia care we can distinguish 3 subsamples:

- The large majority (ca. 65%) does not see an NP during the incidence year.

- A small minority (ca. 7%) sees an NP once during the incidence year, probably for confirmation of diagnosis.

- A large minority (ca. 27%) sees an NP several times in the year before and/or after the incidence quarter. For these continuous utilizers, the NP is obviously taking over a permanent, somehow "primary" care and co-treatment function, a phenomenon not found in the control sample.

The chance to belong to one of these groups is clearly determined by the regional setting and thus probably by differences of supply of specialists in rural vs. urban areas. Reasons and consequences of these regional differences have been discussed in other papers [12,18]

The latter type of contact may reflect the preferences of a segment of the patients (and/or their relatives) and thus may be socially biased [9,17]. Unfortunately, the database does not allow the investigation of factors influencing referral such as educational level. Continuity of specialist care may also be related to the prescription of anti-dementia drugs, as the limited drug budget of the PCP is not charged when the drug is prescribed by a specialist, a question needing further investigation. Both hypotheses are supported by the fact that this continuous visit pattern to the specialist does not take place on the basis of self-referral but on renewed referrals by the PCP in every quarter. On the other hand, this pattern of utilization also reflects the unguided care in the German "free" medical "marketplace" in which every professional recruits his/her own clientele and every patient is entitled to look for the specific offer he/she prefers. In other words, the fact that most of the patients never see a specialist whereas a smaller proportion does so in a continuous manner is certainly not derived from a stepped, collaborative care approach with a specific concept of division of labour between the primary and the secondary care level [19,20]. It should be noted, however, that interface problems between primary and specialist care for patients with dementia are also reported from what appear to be more coordinated healthcare systems such as the UK [21] and Canada [22].

20.5% of all incident cases visited a radiologist in the incidence year. Only very few incident patients are referred by the PCP to radiology or clinical chemistry directly. The PCP probably expects the NP to decide on further diagnostic referrals. The reasons for the relatively low referral rate to radiology by the NP also need further investigation, and this is also the case for the almost zero referral rate to clinical chemistry.

Another point of concern is the low rate of specification of the etiological subtype of dementia by both GPs and NPs and the low rate of correspondence of the etiological subtype between epidemiological studies and the claims data. For example, specific ICD10-codes for Alzheimer's disease (F00/G30) are found in 7.7% of incident persons and codes for vascular dementia in 13.9%, whereas in epidemiological studies an inverse incidence relation (some 3 AD vs.1 VD diagnosis) is found [23]. Also, the fact that more than half of all incident patients are classified as "unspecified" might reflect a lack of eagerness to clarify the etiological subtype. This finding is confirmed by a recent study in southern Germany on the basis of data of another insurance company in which AD was diagnosed in 19% of all dementia patients whereas 53% received a code for "unspecified dementia" [24].

The results of this study on diagnoses and referral rates show important differences between actual ambulatory care and clinical guidelines in Germany. For example, the dementia guidelines of the German Associations for Psychiatry and Psychotherapy and for Neurology strongly (class I) recommend imaging, preferably by CMRT in the course of etiological diagnosis of dementia [25]. The guidelines of the German Association of Family Medicine restrict this recommendation to patients younger than 65 years or in cases where the exclusion of "other causes" is indicated [26]. As for laboratory diagnostics, both guidelines recommend less strongly (class II) a comparable list of analyses in the diagnostic process without any restriction. Such gaps between guidelines and daily practice have been described also for other countries also [16,27-29]. The results from our study raise the question why the gaps between guidelines and daily practice among GPs but also among NPs in the German ambulatory health care system are so important. Also, what can be done to install a discussion about the quality of dementia medical care and how it can be improved? On the one hand, enduring incentives and multi-level projects are needed to increase awareness, acceptance and implementation by professionals [30]. This is admittedly a complicated task as it requires both research and implementation projects. Research should investigate actual barriers in the professions regarding qualification and skills, norms and values and contextual constraints like workload, and payment. Projects should aim at changing working conditions, norms and skills. On the other hand, the large gap between the norm and daily care also poses questions about the applicability of many guidelines [31]. In order to reduce these gaps, experts from actual clinical care should be involved in their development of guidelines in order to ensure that evidence is combined with applicability. Guidelines should not only address issues of clinical management of the individual patient but also forward issues of effective care management, such as division of labour between professionals and questions of co-treatment and referral. In Germany, guidelines including these organizational aspects have been named "systemic" guidelines [32].

Our study has a number of weaknesses but also strengths, extensively described in a previous paper [10]. Its weaknesses are related to problems of validity and timing of the diagnosis of dementia, as discussed above, the size of under- and misdiagnosis and the lack of information on social factors and psychological motives for referrals and for other aspects of utilization of services. Also, the contribution of specialized institutions in diagnosis and care (e.g. geriatric and psychiatric hospitals) is undervalued in this paper. On the other hand, studies based on claims data cover entire patient and provider populations without being impacted by the frequent selection bias in field studies. The reconstruction of complex inter-professional care processes seems hardly possible without such data. Also, the data describe the reality of actual care delivery as they restrict the analysis to those who have been diagnosed as demented within the statutory health insurance system.

## Conclusions

The forthcoming increase of the number of patients with dementia will need a more structured and more effective interface of primary and specialist care and the corresponding referral and co-treatment processes, in order to avoid Feldman's "referral dance" [11]. Guidelines should not only deal with diagnostic procedures and therapeutic options but also consider questions of applicability in daily clinical practice and propose effective organizational models of care provision.

## Competing interests

GG received funding to analyse data of several health insurance companies, for instance the GEK. All other authors declare no conflict of interest.

## Authors' contributions

HvdB, KW and GS conceived the study. GG acquired the data. DK, GS, SS and BW performed the data analysis. HvdB drafted the manuscript. All authors revised the manuscript critically and gave their approval for publication.

## Pre-publication history

The pre-publication history for this paper can be accessed here:

http://www.biomedcentral.com/1472-6963/11/148/prepub
